# Manual Anesthesia Record Keeping at a Tertiary Care Center: A Descriptive Cross-sectional Study

**DOI:** 10.31729/jnma.7117

**Published:** 2021-12-31

**Authors:** Mona Sharma, Dikshya Karki, Saurya Dhungel, Ritika Gautam

**Affiliations:** 1 Department of Anesthesiology, Kathmandu Medical College Teaching Hospital, Sinamangal, Kathmandu, Nepal; 2 Department of Epidemiology, School of Public Health, University of Washington, Seattle, United States of America; 3 KIST Medical College And Teaching Hospital, Imadol, Lalitpur, Nepal

**Keywords:** *anesthesia*, *completion rate*, *intraoperative record*, *medico-legal*

## Abstract

**Introduction:**

Intraoperative record form is one of the cardinal parts of anesthesia practices. Ideally, it should contain complete information about patients under anesthesia and intraoperative events. It serves as valuable information for subsequent patient management, research, or during medicolegal conditions. The objective of this study was to assess the practice and completeness of manual intraoperative anesthesia record keeping.

**Methods:**

A descriptive cross-sectional study was conducted from May 1 to July 31, 2021, in the postoperative ward of Kathmandu Medical College, which is a multispecialty tertiary care center. Approval from the ethical committee of Kathmandu Medical College Teaching Hospital was obtained (Reference: 2603202105) before conducting the study. Convenience sampling was used. The data were entered in Microsoft Excel and statistical analysis was done using Statistical Package for the Social Sciences version 20. Point estimate was done at 95% Confidence Interval and data present in numbers and percentages. We devised forty-two variables, which included demographics, personal identifiers, intraoperative events, anesthesia and airway management, intraoperative parameters, monitoring and medication.

**Results:**

The overall completion rate was 202 (52.59%) (47.6-57.57 at 95% Confidence Interval). Out of 42 variables, the completion rate of 14 variables was less than 50%. Among those were important parameters such as known allergies 94 (24.4%), Body mass index 50 (13%), intraoperative saturation of oxygen 104 (27%), intraoperative electrocardiogram recording 107 (27.8%), total fluid volume administered 45 (11.7%), patient status on transfer 84 (21.8%) had poor completion rate.

**Conclusions:**

Our intraoperative record form shows poor completion rate, which was similar to other studies. many important variables were missing and had incomplete data.

## INTRODUCTION

The anesthesia record is a document that provides information about perioperative care. This provides data on preoperative assessment, anesthesia management, vital parameters, and intraoperative events.^1^

The need for accurate and complete documentation cannot be overemphasized owing to medico-legal aspects, audits, future research and also the quality of care.^2^ The record provides valuable information to other healthcare providers who will be involved in patient care subsequently. ^3^ The intraoperative phase involves anesthesia delivery, monitoring, management of critical events, which can impact proper and complete documentation; especially when patients are unstable. Work stress, manual recording, and practitioner interest are some of the factors, which affect the quality, and practice of documentation.^4^ Most hospitals in Nepal use manual intraoperative anesthesia record keeping.

The objective of this study is to assess the practice and completeness of manual intraoperative anesthesia record keeping.

## METHODS

A descriptive cross-sectional study was conducted from May 1 to July 31, 2021, in the postoperative ward of Kathmandu Medical College, which is a multispecialty tertiary care center. Approval from the ethical committee of Kathmandu Medical College Teaching Hospital was obtained (Ref: 2603202105) before conducting the study. Participants were informed about the nature of the study and informed consent was taken before enrollment, the only exclusion criteria were patient refusal. Since none of our patients refused, all were included. Convenience sampling was used. All record forms filled for elective surgeries performed were included. Based on a previous study,^5^ where the completion rates of records were 50%, the sample size for our study was calculated using the formula:

n = Z^2^ × p × q / e^2^

  = (1.96)^2^ × 0.5 × (1-0.5) / (0.05)^2^

  = 384.16

  = 385

Where,

n= required sample size
p= proportion of completeness of anesthesia record form (50%) according to study the previous^5^
q= 1-p
e= margin of error, 2%
Z= 1.96 at 95% Confidence Interval

The minimal sample size of 385 was calculated. A checklist was created based on our institution record form and policy statement from the Australian and New Zealand College of Anesthetists.^6^ The checklist was finalized after an initial pilot test. The checklist contained questions with three options “yes" for full data entry “incomplete" for partial data entry and “no" for missing data. The data was considered incomplete if full information was missing. For example in the case of date; year, month, and the day had to be written, for data requiring units, for example, weight in kilogram, age in months or year had to be mentioned.

A total of 42 variables were included which were demographics, personnel identifiers, intraoperative events, anesthesia and airway management, intraoperative parameters, monitoring and medication. A 100% completion rate was expected for all the variables. A Completion rate of less than 50% was regarded as a poor result and more than 90% completion was regarded as a good result. The data were entered in Microsoft Excel and statistical analysis was done using Statistical Package for the Social Sciences (SPSS) version 20. Narratives and tables were used to present data in numbers and percentages. Point estimate was done at 95% Confidence Interval and data present in numbers and percentages.

## RESULTS

Out of a total of 385 record forms assessed in this study the overall completion rate was 202 (52.59%) (47.6-57.57 at 95% Confidence Interval). All the patients who had undergone surgery under general and regional anesthesia were included. Among all the record forms studied none had full information filled. The only record that was fully documented was the name of the anesthesia technique i.e. 100%. The records were legible 355 (92.2%) times. The overall completion rate of the chart was only 202 (52.59%).

The patients' name was recorded in full in 377 (98%) charts. It was the most frequently recorded indicator. This was followed by gender 314 (81.6%). The age of the patient was incomplete in 127 (33.0%); these records had no mention of years or months. The inpatient number was filled only in 246 (63.9%) in the record form. Among preoperative details, body mass index (BMI) was filled only in 50 (13%) patients. The mention of known allergies was missing in 291 records (75.6%) (Table 1).

**Table 1:** Intraoperative anesthesia record form completion rate for demographic and other baseline patient information (n=385).

Indicators	Documentation rate n (%)		
	Full data	Incorrect/Partial data	Missing data
Name	377 (97.9)	8 (2.1)	0
Age	258 (67)	102 (26.5)	25 (6.5)
Gender	314 (81.6)	10 (2.6)	61 (15.8)
Weight	194 (50.4)	5 (1.3)	186 (48.3)
Ward/bed no	187 (48.5)	0 (0)	198 (51.4)
Baseline vitals	203 (52.7)	177 (46)	5 (1.3)
Operating theatre number	229 (59.5)	0	156 (40.5)
ASA^*^ status	205(53.2)	0	180 (46.8)
Known allergies	94 (24.4)	0	291 (75.6)

* ASA American Society of Anesthesiologists.

The name of the anesthetist was recorded with full documentation 371 (96.4%), likewise, the name of the procedure was mentioned 362 (94%). However, the total volume of fluids received, mention of critical events was missing in 228 (59.2%), 260 (67.6%), 234(60.8%) records respectively. During the study duration, 258 cases were done under regional anesthesia. The site of regional anesthesia was fully documented in 157 (60.8%) and completely missing in the rest 101 (39.2%). Among 127 patients receiving general anesthesia, full documentation of the type of airway device used was done in 77 (60.6%) records, laryngoscopy grade was mentioned in full data in 118 (92%) (Table 2).

**Table 2:** Intraoperative anesthesia record form completion rate for intraoperative events and anesthesia technique.

Indicators	Documentation rate n (%)		
	Full data	Partial data	Missing data
Date of surgery	275 (71.4)	5 (1.3)	105 (27.3)
Name of surgeon	252 (66.5)	128 (33.2)	5 (1.3)
Name of anesthetist	371 (96.4)	9 (2.3)	5 (1.3)
Name of the procedure	362 (94)	18 (4.7)	5 (1.3)
Type &size of airway device used	77 (60.6)	47 (37)	3 (2.4)
Laryngoscopy grade	118 (92.1)	0	9(7.9)
Ventilator mode and parameters	48 (37.7)	54 (42.5)	25 (19.8)
Fresh gas flow	53 (41.7)	23 (18.1)	51 (40.2)
Open or closed circuit	60 (47.2)	27 (21.2)	31.6 (30.7)
Fluid type	354 (91.1)	8 (2.1)	23 (6)
Total fluid volume	45 (11.7)	80 (20.8)	260 (67.6)
Total time for surgery	176 (45.7)	143 (37.1)	66 (17.1)
Total time for anesthesia	136 (35.3)	157 (40.8)	92 (24)
Patient's status on transfer	84 (21.8)	52 (13.5)	249 (64.7)
Mention of intraoperative critical events	119 (30.9)	32 (8.3)	234 (60.8)

The drug dosage was mentioned with full documentation in 347 (90.1%) of cases; however, the drug route was missing in all cases. Likewise, the time at which the drug was given was missing in 360 (93.5%). An extremely important detail of intraoperative vitals like the saturation of oxygen (SPO_2_) was missing in 212 (55.1%) of records. Documentation of care of pressure points, blood loss was completely missing in 257 (66.8%), 208 (54%) respectively (Table 3).

**Table 3:** Intraoperative anesthesia record form completion rate for intraoperative monitoring and medications.

Indicators	Documentation rate n (%)		
	Full data	Incorrect/Partial data	Missing data
Patient position	258 (67)	0	127 (33)
IV/arterial/CVP line size	288 (74.8)	33 (8.6)	64 (16.6)
^†^IV/arterial/CVP line cannulation site	303 (78.7)	59 (15.3)	23 (6)
Drug dosage	347(90.1)	0	38 (9.9)
Drug route	0	0	385 (100)
Drug timing	25 (6.5)	0	360 (93.5)
Intraoperative HR	237 (61.6)	132 (34.3)	16 (4.2)
Intraoperative BP	293 (76.1)	76 (19.7)	16 (4.2)
Intraoperative SPO_2_	104 (27)	69 (17.9)	212 (55.1)
Intraoperative rhythm (ECG)	107 (27.8)	120 (31.2)	158 (41)
Blood loss	107 (27.8)	70 (18.2)	208 (54)
Care of pressure points	128 (33.2)	0	257 (66.8)
Urine output	129 (33.5)	0	256 (66.5)
Monitors used	174(45.2)	148 (38.4)	63 (16.4)

† IV intravenous, CVP central venous pressure

## DISCUSSION

None of the charts were filled completely. The overall completion rate of the chart was only 202 (52.59%). Sub par documentation of manual record system has been documented in many previously conducted studies.^5,7–10^ Incomplete notes and abbreviations are a source of weak defense during litigations. Apart from that, information derived from record forms is of extreme value to subsequent practitioners who may give anesthesia services to the patient.^2,3^

Less than 50% completion rate was seen for many variables. Only 50.4% documentation of weight and missing data of BMI suggests that inaccurate dosage of drugs, ventilation parameters, airway equipment size and fluid management. This could result in poor quality of anesthesia management. Our finding correlates with other studies conducted.^5^

Demographic data like name, age and gender had more than 80% completion rate in this study. Some studies conducted in Africa have published results where these parameters had a poor completion rate, especially for the name of the patient.^5,7^ This could be because of long names in the African continent. However, inpatient number and ward or bed number were fully recorded only in 63.9% and 48.5%. These can lead to misidentification of patients with similar names. Allergic reactions in the intraoperative setting can be devastating especially in case of late diagnosis. Therefore, patients with known allergies have to be identified. However, in our findings, only 24.4% of records form had mentioned if the patient had any allergies. Catastrophic complications can occur when vital information as history of allergic reactions is missing in the record form.

Intraoperative vital parameters recorded in our study were heart rate, rhythm, oxygen saturation and blood pressure recorded every 5 minutes. The complete record of these parameters was poor especially for SPO_2_ and rhythm recorded by electrocardiograph (ECG), which was 27% and 27.8% respectively. This implies late detection and management of critical events, which in turn affects overall patient outcome. Such similar findings are depicted in other studies performed by Gebremedhn, et al.^8^ and Raff, et al.^9^

Drug dosing is of extreme importance in anesthesia, many factors play into action such as age, weight, comorbidities etc. In this study drug dose was fully mentioned in 90.1% of cases, however, the completion rate for drug timing was only 6.5% and the drug route was missing in all record forms. In previous studies done have shown a higher percentage of recording rates for these variables. This could be because of a structured intraoperative form, that we use, drug route and timing is not mentioned, nevertheless, it should have been recorded.^5,8^

The completion rate of variables like ventilator parameters, fresh gas flow, total fluid given, urine output, check of pressure points, intraoperative critical events, status of patient on transfer were poor that is below 50%. This poor documentation is sub-par of expected completion rate. This could signify poor quality of perioperative anesthesia services. These data could be against anesthesia service providers, as something that is not written did not happen. Therefore, where litigation claims are made, these under documented forms give us a lower hand.

However, variables such as name of the anesthetist, name of the procedure, laryngoscopy grade, fluid type and drug dosage was fully completed in more than 90% record forms. Similar high documentation rate has been recorded in studies conducted previously.^8-10^

Although many studies have included postoperative orders in anesthesia form, in our center we record postoperative order in another form, hence was not included in this study.

Our findings suggest that there are significant deficiencies in the adequate documentation of anesthetic record form. This has been shown mainly in recording of baseline and intraoperative vitals namely saturation of oxygen, blood loss, care for pressure points, mention of critical events, drug route and timing. These findings suggest that our intraoperative record form serves as a poor document for research, medico legal document and data for future reference. There are reports suggesting that critical events or complications that occur are intentionally not recorded for the fear of litigation, blame by colleagues and disciplinary action by the hospital management.^11^

A study conducted in 2015 has shown that when awareness is created about the importance of anesthesia record form by teaching and regular audit can improve the overall completion rate of the record form.^12^

According to new studies limitation of manual recording, can be over come by computerized record-keeping; as it has certain advantages like accurate record of intraoperative vital parameters, better quality of care and regulatory compliance.^13,14^

This study has limitation as it was performed at one teaching hospital and does not reflect the practices at other hospitals. Our study does not include preoperative chart, which contains pre-anesthetic evaluation of the patient. Nor does our study include postoperative order. Besides these, we have only included elective surgeries. Studies have shown that completion rate of intraoperative record form for emergency surgeries are poor.^1,10^

## CONCLUSIONS

Our intraoperative record form shows a poor completion rate, many important variables were missing, and had incomplete data. Important parameters like the history of allergies, intraoperative saturation of oxygen and electrocardiogram recording, information about drugs route and intraoperative total fluids, patients' status on transport from operating theatre had less than 50% completion rate, i.e. poor result.
